# Investigation of the mechanistic impact of CBL0137 on airway remodeling in asthma

**DOI:** 10.1186/s12890-025-03596-y

**Published:** 2025-03-20

**Authors:** Zhiheng Huang, Liangxian Li, Bingxi Zhang, Dong Yao, Bo Xiao, Biwen Mo

**Affiliations:** 1 Department of Respiratory and Critical Care Medicine, Guangxi Clinical Research Center for Diabetes and Metabolic Diseases, Guangxi Health Commission Key Laboratory of Glucose and Lipid Metabolism Disorders, The Second Affiliated Hospital of Guilin Medical University, Guilin, 541199 China; 2 Chest Hospital of Guangxi Zhuang Autonomous Region, Liuzhou, 545005 China; 3 Guangxi Key Laboratory of Brain and Cognitive Neuroscience, Guilin Medical University, Guilin, 541000 China; 4 Department of Pulmonary and Critical Care Medicine, The Laboratory of Respiratory Disease, Affiliated Hospital of Guilin Medical University, Guilin, 541001 China; 5 Guangxi Key Laboratory of Metabolic Reprogramming and Intelligent Medical Engineering for Chronic Diseases, the Key Laboratory of Respiratory Diseases，Education Department of Guangxi Zhuang Autonomous Region, Guilin Medical University, Guilin, 541001 China; 6 Laboratory of Basic Research on Respiratory Diseases, Guangxi Health Commission, Affiliated Hospital of Guilin Medical University, Guilin, 541001 China

**Keywords:** Airway remodeling, Airway smooth muscle cells, Asthma, CBL0137

## Abstract

**Background:**

Bronchial asthma, a chronic inflammatory airway disease, is characterized by airway remodeling, including thickening of the airway smooth muscle layer, primarily due to abnormal proliferation of airway smooth muscle cells (ASMCs). CBL0137 (Curaxin-137 hydrochloride), a histone chaperone facilitate chromatin transcription (FACT) inhibitor, has demonstrated anti-tumor properties, including inhibition of proliferation, promotion of apoptosis, and increased autophagy. However, its effects on ASMCs and airway remodeling remain unexplored.

**Methods:**

Asthma models were established using ovalbumin (OVA) in female C57BL/6 J mice, with therapeutic interventions using CBL0137 and budesonide. Lung tissues were analyzed using Hematoxylin and eosin (H&E), PAS, Masson’s trichrome, and α-SMA immunofluorescence staining. ASMCs extracted from Sprague–Dawley rats were cultured in vitro experiments, with phenotypic changes assessed via flow cytometry. Gene and protein expressions were analyzed using RT-PCR and Western blotting.

**Results:**

CBL0137 significantly reduced airway resistance, goblet cell proliferation, alveolar collagen deposition, and airway smooth muscle layer thickening in asthmatic mice. In vitro, CBL0137 inhibited ASMC proliferation and induced apoptosis, downregulating cyclin-B1, Cdc2, and Bcl-2 while upregulating caspase-3.

**Conclusions:**

CBL0137 mitigates airway remodeling of asthmatic mice by modulating ASMC proliferation and apoptosis, presenting a potential therapeutic strategy for asthma treatment.

**Supplementary Information:**

The online version contains supplementary material available at 10.1186/s12890-025-03596-y.

## Background

Bronchial asthma is a prevalent chronic inflammatory disease of the airways [1]. According to the Global Initiative for Asthma (GINA), approximately 300 million individuals worldwide are affected by asthma, and this figure is projected to exceed 400 million by 2025 [2]. Asthma imposes a significant economic burden on both society and individuals and has emerged as a chronic condition that seriously endangers global health [3]. Investigating its pathogenesis and identifying effective interventions remain major academic priorities.

Chronic airway inflammation is a key factor in the pathogenesis of asthma, with airway remodeling being an inevitable consequence of prolonged inflammation [4]. This remodeling is driven by airway damage and impaired repair mechanisms under the influence of various inflammatory factors. Pathological changes include alterations in airway epithelial morphology, astrocyte hyperplasia, collagen deposition in lung parenchyma, smooth muscle cell proliferation, extracellular matrix deposition, and neovascularization [5]. Notably, the thickening of the airway smooth muscle (ASM) layer is a hallmark of asthma [6], with ASM cells (ASMCs), playing a pivotal role in airway remodeling by actively participating in its progression [7]. Consequently, targeting ASMC dysfunction is a focus of current asthma research [8–10].

CBL0137, a curacin compound and an inhibitor of histone chaperone-FACT, exhibits potent antiproliferative, proapoptotic, and autophagy-promoting effects on tumor cells through various mechanisms [10]. Studies have demonstrated that CBL0137 inhibits hepatocellular carcinoma cell proliferation by modulating P53 and nuclear factor-kappa B (NF-κB) pathways and apoptosis-related proteins, such as PARP and caspases [11]. Similar effects have been observed in ovarian cell carcinoma and non-Hodgkin’s lymphoma (B-NHL) [12, 13]. However, the impact of CBL0137 on ASM remains unexplored. Whether CBL0137 can mitigate airway remodeling in asthma by influencing ASMC proliferation and apoptosis remains unclear.

In preliminary investigations, our group found that CBL0137 exhibits cytotoxic effects on rat primary ASMCs, with cell viability decreasing as drug concentration and exposure duration increase. Building on this, we aim to evaluate the effects of CBL0137 on asthma-related airway remodeling using an ovalbumin (OVA)–induced mouse model. The study involves assessing lung function, histopathological changes, and special staining to determine whether CBL0137 affects ASM thickening. Additionally, we will explore the underlying mechanisms by examining the effects of CBL0137 on ASMC proliferation and apoptosis, aiming to identify novel therapeutic agents for asthma-related airway remodeling.

## Materials and methods

### Animal models

Female C57BL/6 J mice (6–8 weeks old, 18–22 g) were procured from the Hunan Laboratory Animal Center (Permit No. SCXK 2019–0004). The mice were housed in a specific pathogen-free (SPF) environment, with sterilized cages, bedding, and feed. Cages were changed twice weekly, and mouse weights were recorded weekly. This study was approved by the Laboratory Animal Ethics Committee of Guilin Medical University (Approval No. GLMC202308001).

Twenty-five female C57BL/6 J mice were randomly divided into five groups, with five mice per group: phosphate-buffered saline (PBS) control, OVA only, OVA + CBL0137 (15 mg/kg), OVA + CBL0137 (30 mg/kg), and OVA + budesonide. Sensitization was performed on Days 1, 14, and 21 by intraperitoneal injection of 0.1 mg of OVA (Sigma‒Aldrich A5503) dissolved in double-distilled water and mixed thoroughly with 100 μL of aluminum hydroxide (IA5810, Solarbio).

### Stimulation period

**Fig. 1:**
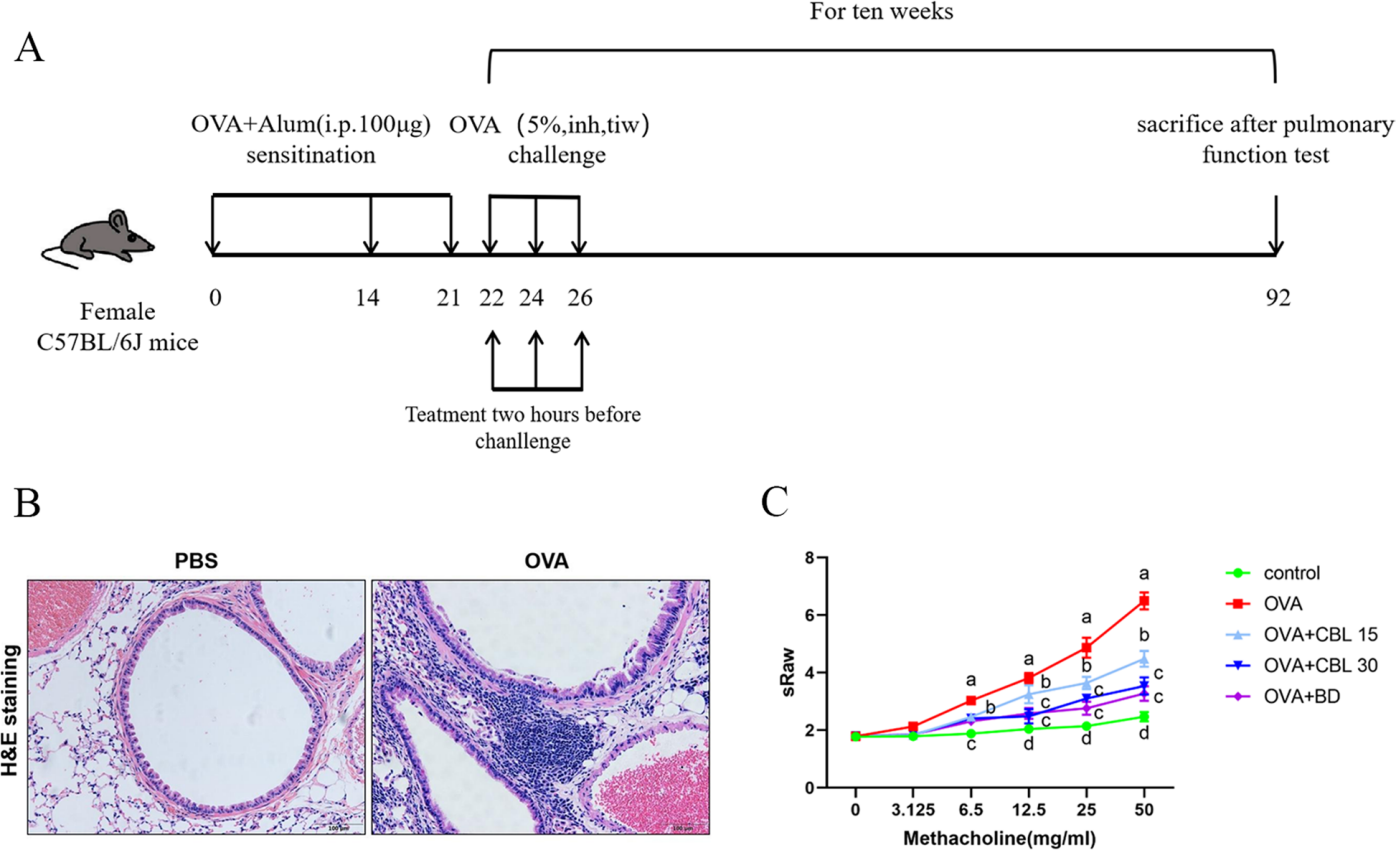
Effect of CBL0137 on airway resistance in asthmatic mice. **A** Schematic representation of the asthmatic mouse model. **B** Hematoxylin and eosin (H&E) staining of lung tissue. **C** Evaluation of functional airway resistance in the pulmonary system of mice. Mice were exposed to nebulized acetylcholine (0, 3.125, 6.25, 12.5, 25, and 50 mg/mL) for 3 min, and airway resistance was measured in real time. Linear plots were generated to depict resistance changes. Statistical comparisons between consecutive groups at the same acetylcholine concentration revealed significant differences (a vs. b, b vs. c, c vs. d; ^*^p < 0.05)

CBL0137 (Aladdin, 44,519,124, Shanghai, China) was administered intraperitoneally at doses of 15 mg/kg or 30 mg/kg on the 22nd day of each week, specifically on Days 1, 3, and 5. In the positive control group, 2 mg of budesonide (China Tianqing Speed Fluid) was delivered via nebulized inhalation, followed by administration of 5% OVA through nebulized inhalation 2 h later for 30 min (positive control group: OVA + budesonide). For the control group, PBS was used instead of OVA. On the 92nd day, after lung function measurements, lung tissues were harvested for assay (Fig. 1A).

Isoflurane, a safe, efficient, and readily accessible anesthetic commonly used in animal experiments, was selected for this study. The procedure was approved by the Laboratory Animal Ethics Committee of Guilin Medical University. Mice were anesthetized, their whiskers were trimmed, and blood specimens were collected via eyeball removal into 1.5-mL EP tubes. The mice were subsequently fixed on a dissection table, and the abdomen was opened to expose the abdominal aorta, which was severed for blood drainage. The thoracic cavity was opened to expose the lungs and trachea. A small incision was made in the main trachea using ophthalmic scissors, and a 1-mL syringe needle with a plastic cannula was inserted and secured with a cotton thread ligature. The left pulmonary hilar was clamped using hemostatic forceps, 500 µL of PBS was infused into the right lung, and the lavage fluid was collected. After lavage, the procedure was repeated for the left lung using 4% paraformaldehyde. The left lung was then excised and preserved in 4% paraformaldehyde, while the right lung was divided into four lobes, which were stored in specimen tubes. Mouse carcasses were disposed of according to ethical guidelines.

Previous studies demonstrated that CBL0137 exerts anti-tumor effects in a mouse model of pancreatic ductal adenocarcinoma when administered via tail vein injection at 60 mg/kg once weekly [13]. However, the participants in this study had not yet mastered the technique of tail vein injection in mice. Consequently, a safer and more easily implementable intraperitoneal injection method was selected. Since the drug was administered three times a week for 10 weeks, the cumulative dosage exceeded that used in previous tumor studies. Therefore, the single dose was less than 60 mg/kg. Considering the maximum dissolution volume of CBL0137 and the maximum intraperitoneal injection volume of 200 μL in mice, the experimental dosages were established as 15 and 30 mg/kg.

### Behavioral observations in mice

On the 92nd day of the 10th week of stimulation, mice were individually placed in clean cages under natural conditions. They were observed for signs of restlessness, frequent nose touching, and thoracic tremors indicative of respiratory distress during rest. Each mouse was monitored for 10 min, and the frequency of nose touching was recorded per unit of time.

### Mouse lung function measurement

Lung function was assessed after 10 weeks of stimulation. Acetylcholine excitation solutions were prepared with PBS at concentrations of 0, 3.125, 6.25, 12.5, 25, and 50 mg/mL. Airway resistance was measured using a lung function meter while the mice remained awake. Each mouse was fixed in a sealed chamber, and the respiratory curve was observed after the instrument was activated. Once the curve stabilized, 50 μL of the excitation solution was added to the dosing compartment. The nebulization preparation time was 1 min, followed by 3 min of nebulization and inhalation. Airway resistance values were recorded in real-time. This procedure was repeated for each mouse across all acetylcholine concentrations.

### Lung tissue sample collection

The mice were euthanized and secured on a dissection table. The abdominal cavity was opened, and the abdominal aorta was severed. Blood was expelled from the lungs by compressing the chest cavity. The thoracic cavity was dissected to expose the main trachea. A small diagonal incision was made below the thyroid cartilage using ophthalmic scissors. A 5-gauge needle with a rubber cannula (attached to a 1-mL syringe) was inserted into the trachea and secured with surgical sutures. The right lung portal was clamped with hemostatic forceps, and 4% paraformaldehyde was injected into the left lung until it was sufficiently distended. The left lung portal was then ligated, excised, and preserved in 4% paraformaldehyde. The right lung was removed, divided into four lobes, and stored at − 80 °C for freezing. The mouse carcasses were placed in designated disposal bags and sent for proper waste treatment.

### Enzyme-linked immunosorbent assay

Collected blood samples were left to stand for 2 h and then centrifuged to obtain the upper serum layer. The same method was applied to obtain the supernatant from alveolar lavage fluid. Enzyme-linked immunosorbent assay (ELISA) was conducted according to the laboratory manual. The average optical density (OD) values of standard and sample duplicate wells were calculated after subtracting the blank well OD values as calibration. A standard curve was generated using a four-parameter logistic function, with concentration as the horizontal axis and OD values as the vertical axis.

### Hematoxylin–eosin staining

Tissue sections were initially soaked in distilled water and stained with hematoxylin solution for 5 min. The sections were then treated with acidic water or ammonia for 5 s each, rinsed with distilled water, and washed under running water for 60 min. For dehydration, the sections were sequentially placed in 70% and 90% ethanol for 10 min each. Eosin stain was added for 3 min during the dehydration process. Following dehydration, the sections were treated with xylene to enhance transparency. Drops of mounting medium were applied to the sections, which were then covered with coverslips and allowed to dry slightly before use.

For data collection, intact airways were examined under a 200 × microscope [14, 15]. Airway inflammation was scored using a blinded method as follows: grade 0 (no inflammatory cells observed), grade 1 (occasional inflammatory cells observed), grade 2 (bronchial tubes surrounded by one to three layers of inflammatory cells), grade 3 (bronchial tubes or blood vessels surrounded by four to five layers of inflammatory cells), and grade 4 (bronchial tubes or blood vessels surrounded by more than five layers of inflammatory cells).

### Periodic acid–Schiff staining

The samples were dewaxed as follows: xylene I, II, and III were each used for 5 min; anhydrous ethanol, 95% ethanol, and 75% ethanol were applied for 1 min each; and distilled water was used for 5 min. Next, 50 µL of Alcian blue staining solution was applied at room temperature for 20 min. The samples were then rinsed with distilled water for 2 min, with this step repeated three times. Oxidation with an oxidizing agent was performed 5 min after rinsing with tap water, followed by immersion in distilled water, which was repeated twice. Subsequently, 50 µL of Schiff staining solution was applied for 20 min at room temperature, after which the samples were rinsed with running water. The nuclei were stained using hematoxylin solution for 2 min, followed by rinsing with running water. After immersion in an acidic differentiation solution for 5 s, the samples were rinsed again. The nuclei were stained with Scott’s blue solution for 3 min and rinsed for an additional 3 min.

Under the microscope, purple-blue staining indicates cup-shaped cells, while nuclei appear light blue. Data collection: Intact airways were selected under 200 × magnification, and cup-shaped cell hyperplasia was assessed based on the method by Padrid et al. [15]. Pathological changes were quantified using a modified 5-point scale (0–4) based on the percentage of cup-shaped cells in the epithelium: grade 0 (no cup-shaped cells observed), grade 1 (< 25% of epithelial cells are cup-shaped), grade 2 (25%–50% of epithelial cells are cup-shaped), grade 3 (51%–75% of epithelial cells are cup-shaped), and grade 4 (> 75% of epithelial cells are cup-shaped). At least eight bronchioles were counted per slide, and the mean hyperplasia score was calculated for each mouse.

### Masson’s trichrome staining

The tissue sections were dewaxed to a liquid state and immersed in ferric hematoxylin staining solution for 10 min. Following this, the samples were rinsed with running water. If overstaining occurred, hydrochloric alcohol was used to correct it. The sections were then treated with Masson bluing solution for 5 min and rinsed again. Afterward, the sections were immersed in rejuvenated magenta staining solution for 10 min, washed with distilled water for 1 min, and treated with a weak acid working solution (1% weak acid to 2% distilled water) for 1 min.

Subsequently, the samples were rinsed with 1% phosphomolybdic acid solution for 2 min and then washed with the weak acid solution for 1 min. The sections were directly immersed in aniline blue staining solution for 2 min. After washing and rinsing with the weak acid solution, the samples were rapidly dehydrated in 95% ethanol. This was followed by three rounds of dehydration using anhydrous ethanol for 10 s each. Finally, the sections were immersed in xylene for 2 min, a process repeated three times, and sealed with neutral gum.

For the staining results, under the microscope, collagen fibers appear blue, muscle fibers red, and nuclei black–blue. Data collection: Intact airways were analyzed under 200 × magnification. The collagen fiber deposition area (blue) was boxed using graphic software, and the area (µm^2^) was measured. The airway circumference (µm) was also measured, and the collagen deposition percentage was calculated using the formula: perimeter area (µm^2^)/airway circumference (µm) [16]. A minimum of eight fine bronchioles were assessed per slide, and the mean percentage of collagen fibers was calculated for statistical analysis.

### Immunofluorescence staining

The samples were dewaxed by immersion in xylene I, II, and III for 20 min each. This was followed by sequential washing in anhydrous ethanol I, II, 90% ethanol, and 80% ethanol for 10 min each. The samples were then rinsed with distilled water for 5 min.

For antigen retrieval, slides were placed in a metal box containing EDTA antigen repair buffer (pH 8.0). The box was heated in an electric cooker at high temperature for 3 min, then allowed to cool for 10 min before reheating for another 3 min. Afterward, the slides were allowed to cool naturally and rinsed with PBS (pH 7.4) on a shaking table for 5 min, repeated three times.

The sections were soaked in paraffin-containing PBS for 3 min, repeated three times. Then, 200 µL of 5% BSA was applied to each section and incubated for 30 min. The primary antibody (diluted 1:1000 in PBS) was added dropwise (50 µL per section), and the samples were incubated overnight at 4 °C. Since the primary antibody was self-fluorescent, secondary antibody incubation was unnecessary.

After washing with PBST for 3 min (repeated three times), diluted DAPI was added, and the samples were incubated at room temperature for 10 min. The sections were then sealed with 50 µL of antifluorescence attenuator sealer and covered with coverslips.

The staining results were as follows: DAPI-stained nuclei exhibited blue fluorescence, while positive sites showed red fluorescence. Data acquisition: Complete airways were selected under 200 × magnification, and images were processed using ImageJ software. The fluorescence channels were split, and the average fluorescence intensity (mean) was calculated using the formula: sum of fluorescence intensity (IntDen)/area (Area).

### Isolation and culture of rat ASMCs

Female SPF-grade Sprague–Dawley rats (260–300 g) were procured from the Hunan Laboratory Animal Center. After anesthesia and sterilization, lung tissues were excised under aseptic conditions, and bronchial tubes were washed with precooled D-Hanks solution (4 °C). The bronchial tubes were minced, and 0.2% collagenase type I was added for 1 h. The centrifuged bronchial tissues were resuspended in a complete medium containing 20% fetal calf serum and cultured in 25-T flasks for 1 week to allow adherent growth.

Wall-adherent cells were digested with 0.25% trypsin–EDTA and purified by differential wall adherence. Smooth muscle cells were identified through immunostaining with anti-α-smooth muscle antibody (α-SMA; 1:100, Sigma). Cells from passages 3 to 5 were used for subsequent experiments.

### Cell counting Kit-8 assay

ASMCs were seeded into 96-well plates (2 × 10^3^ cells/well). After 24 h of attachment, CBL0137 (0, 0.1, 0.2, 0.4, 0.8, 1.6, or 3.2 μM) was added, and the mixture was incubated for 24, 48, or 72 h. Cell viability was assessed using a Cell Counting Kit-8 (CCK-8) assay according to the manufacturer’s instructions. All experiments were performed three times.

### EdU assay

ASMCs were cultured on sterile coverslips placed in 24-well plates at a density of 2 × 10^4^ cells per well. After 24 h of incubation, varying concentrations of CBL0137 (0, 0.1, 0.2, 0.4, 0.8, 1.6, or 3.2 μM) were added, and the cells were incubated for 24, 48, or 72 h. Cell proliferation was assessed using the BeyoClick™ EdU Cell Proliferation Kit with Alexa Fluor. The coverslips were observed under an upright fluorescence microscope (Olympus BX53, Tokyo, Japan). All experiments were conducted in triplicate.

### Cell cycle detection

ASMCs were seeded into 6-well plates at a density of 4 × 10^5^ cells per well. Following 24 h of incubation, CBL0137 (0, 0.5, 1, 1.5, 2, 2.5, or 3 μM) was added. Cells were harvested, digested with 0.25% trypsin (without EDTA), washed with ice-cold PBS, and fixed in 70% ethanol at − 20 °C overnight. The fixed cells were treated with RNase A and stained with propidium iodide (PI) solution for 15 min. The cell cycle distribution was analyzed using flow cytometry, and data were processed with FlowJo 10 software. All experiments were performed in triplicate.

### Cell apoptosis detection

ASMCs were cultured into 6-well plates at a density of 4 × 105 cells per well. After 24 h, CBL0137 (0, 0.5, 1, 1.5, 2, 2.5, or 3 μM) was added, and the cells were incubated for 48 h. Apoptosis was assessed using an Annexin V-FITC/PI Apoptosis Detection Kit following the manufacturer’s instructions. Each experiment was repeated three times.

### Quantitative reverse transcription polymerase chain reaction assay

ASMCs were seeded into 6-well plates at a density of 4 × 105 cells per well. After 24 h, CBL0137 (0, 0.5, 1, 1.5, 2, or 2.5 μM) was added, and the cells were incubated for 48 h. Total RNA was extracted using TRIzol reagent, and 0.8 μg of RNA was reverse transcribed into cDNA using a dsDNase kit. Quantitative reverse transcription polymerase chain reaction (qRT-PCR) was performed using MonAmp™ SYBR® Green qPCR mix and a CFX96 Real-Time System (Shanghai, China).

Primers for Cyclin B1 ((forward: ACAGTCAAGAATACCCCTCA; reverse: GCTCATCCAGTTCCACCT), Cdc2 (forward: GACAAAGGAACAATCAAACT; reverse: TACCACAGCGTCACTACC), Bc1-2 (forward: CACAGAGGGGCTACGAGT; reverse: GGCTGGAAGGAGAAGATG), Caspase-3 (forward: TGGACTGCGGTATTGAGAC; reverse: CGGGTGCGGTAGAGTAAGC), and GAPDH (forward: GACATGCCGCCTGGAGAA; reverse: AGCCCAGGATGCCCTTTAGT) were synthesized by Gene Create (Wuhan, China). Relative expression levels were calculated using the ΔΔCT method and normalized to GAPDH expression. All experiments were conducted in triplicate.

### Western blotting

ASMCs were cultured in 6-well plates at a density of 4 × 105 cells per well. After 24 h, CBL0137 (0, 0.5, 1, 1.5, 2, or 2.5 μM) was added, and the cells were incubated for 48 h. Total protein was extracted using RIPA lysis buffer and quantified with a BCA protein assay kit. Proteins were separated via SDS-PAGE, transferred onto PVDF membranes, and blocked with 5% nonfat milk at room temperature. Membranes were incubated with primary antibodies against Cyclin B1, Cdc2, Bc1-2, Caspase-3 (1:500; Santa Cruz Biotechnology), GAPDH, and Tubulin (1:10,000; Affinity Biosciences) for 1 h. Following washes with TBST, membranes were incubated with goat anti-mouse IgG secondary antibody (1:5000; ZSGB-BIO) for 2 h. Signals were developed using an ECL reagent, and protein levels were quantified using ImageJ software. Experiments were conducted in triplicate.

#### Statistical analysis

All experiments were performed in triplicate, and the data are presented as the means ± standard deviations (*SD*s). A one-way analysis of variance (ANOVA) was conducted for multiple group comparisons using GraphPad Prism 8. Statistical significance was set at *p* < 0.05 (^*^p < 0.05; ^**^p < 0.01), indicating significant differences between groups. Data were imported into GraphPad Prism 8, which was used for both statistical analysis and graph generation.

## Results

### CBL0137 reduces airway resistance in asthmatic mice

An asthma mouse model was successfully established (Fig. 1A). Compared with the blank control group, asthmatic mice exhibited reduced locomotion, increased irritability, and a significantly higher frequency of nasal touches during the resting state. Pronounced thoracic fibrillation was observed during upright respiration, accompanied by audible forced breathing sounds. Histological analysis of H&E-stained lung tissue revealed substantial inflammatory cell infiltration in the alveoli, bronchial lumen, walls, and surrounding vasculature of the OVA group mice. Additionally, these mice exhibited increased bronchial mucosal folding and thickened smooth muscle layers (Fig. 1B), confirming the successful induction of asthma.

Pulmonary function tests were conducted to assess airway resistance in asthmatic mice, which were nebulized with acetylcholine at concentrations of 0, 3.125, 6.25, 12.5, 25, and 50 mg/mL. Real-time monitoring using a lung function meter revealed that airway resistance increased with acetylcholine concentration in all groups (Fig. 1C). At acetylcholine concentrations ≥ 6.25 mg/mL, airway resistance in the OVA and treatment groups was significantly higher than that in the blank control group (*p* < 0.05). Notably, airway resistance in the CBL0137-treated groups was lower than that in the OVA group. While no significant differences were observed among the three treatment groups at 6.25 mg/mL, at concentrations ≥ 12.5 mg/mL, airway resistance in the CBL0137 low-dose group (15 mg/kg) was significantly higher than that in the CBL0137 high-dose group (30 mg/kg) and the budesonide group (*p* < 0.05). However, no significant difference was noted between the CBL0137 high-dose and budesonide groups.

### CBL0137 inhibits airway remodeling in asthma

Lung function analysis indicated that CBL0137 effectively reduced airway resistance in asthmatic mice. To elucidate the underlying mechanism of this reduction, we investigated key factors related to airway remodeling: increased mucus secretion from the airway epithelium, interstitial fibrin deposition, and thickening of the ASM layer.

**Fig. 2:**
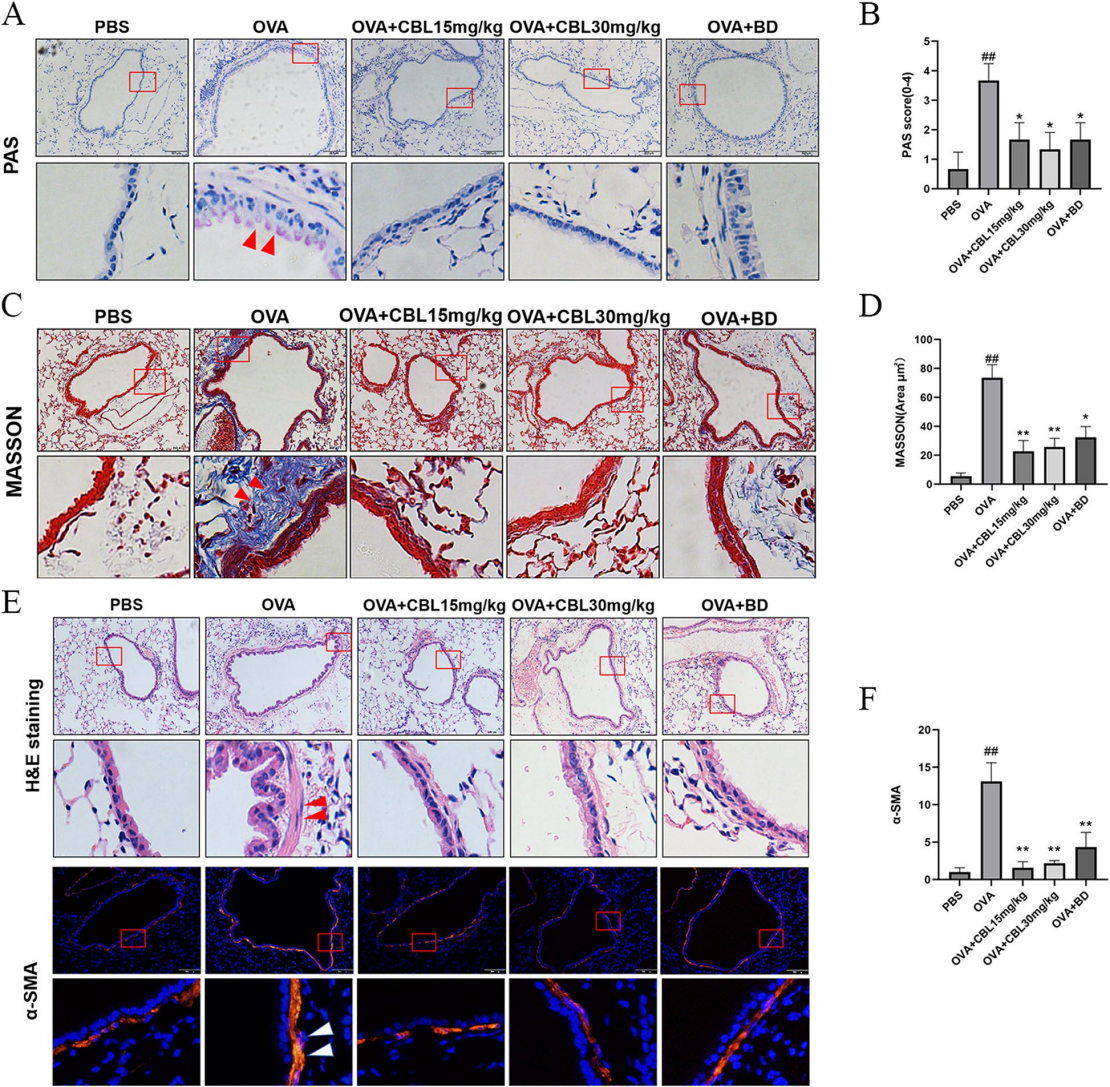
CBL0137 inhibits airway remodeling in asthma. **A** PAS staining of mouse lung tissue sections at 200 × and 400 × magnifications. **B** Statistical analysis of PAS staining scores (^*^model group vs. blank control group: ^##^*p* < 0.01; low-dose CBL group vs. modeling group: ^**^p < 0.01; high-dose CBL group vs. modeling group: ^*^p < 0.05; BD group vs. modeling group: ^*^p < 0.05). **C** Masson’s trichrome staining of mouse lung tissue sections at 200 × and 400 × magnifications. **D** Statistical analysis of collagen fiber deposition area (model group vs. blank control group: ^##^*p* < 0.05; low-dose CBL group vs. model group: ^**^p < 0.01; high-dose CBL group vs. model group: ^**^p < 0.01; BD group vs. model group: ^*^p < 0.05). **E** H&E staining and α-SMA immunofluorescence staining of mouse lung tissue sections at 200 × and 400 × magnifications. **F** Statistical analysis of mean α-SMA immunofluorescence intensity (^*^model group vs. blank control group: ^##^*p* < 0.01; low-dose CBL group vs. model group: ^**^p < 0.01; high-dose CBL group vs. model group: ^**^p < 0.01; BD group vs. model group: ^*^p < 0.01)

Periodic acid–Schiff staining was performed on lung tissue sections from the experimental groups (Fig. 2A). Scoring was conducted according to established criteria: the blank control group scored 0 or 1, the OVA-induced asthma group scored 3 or 4, and the medicated group scored 1 or 2. The asthma model group displayed significantly increased blue-purple staining in the airway epithelium compared with the blank control group, indicating elevated mucus secretion. In contrast, the medicated groups exhibited reduced staining compared with the OVA group, with no significant differences observed between these groups and the blank control group. Additionally, no statistically significant differences were found in pairwise comparisons among the three medicated groups (Fig. 2B).

Masson’s trichrome staining was used to assess collagen fiber deposition in the lung tissue (Fig. 2C). The asthma model group showed substantial collagen deposition in the endothelial basal layer of the airways and the paratracheal alveolar tissue compared with the blank control group. Treatment with CBL0137 significantly reduced collagen deposition in all medicated groups relative to the asthma model group, although collagen levels remained higher than those in the blank control group. No significant differences were detected in pairwise comparisons among the medicated groups (Fig. 2D).

Hematoxylin and eosin staining, along with α-smooth muscle actin (α-SMA) immunofluorescence staining, was employed to evaluate ASM layer thickening (Fig. 2E). H&E staining revealed a pronounced thickening of the ASM layer in the asthma model group compared to the blank control group. Higher magnification showed proliferation and hypertrophy of smooth muscle cells, elongated nuclei, and stacked layers of thickened smooth muscle around the airways. α-SMA staining confirmed a significant increase in fluorescence intensity, indicating elevated α-SMA expression in the asthma model group. However, treatment with CBL0137 led to reduced smooth muscle layer thickness and α-SMA fluorescence intensity in all medicated groups compared to the asthma model group. Notably, no significant differences were observed between the CBL0137 treatment group and the BD treatment group in terms of smooth muscle thickness or α-SMA expression (Fig. 2F).

To further investigate the potential effects of CBL0137 on airway remodeling, we measured the levels of asthma-associated inflammatory factors, including interleukins (IL-4, IL-5, and IL-13), immunoglobulin E (IgE), and immunoglobulin G (IgG), in serum and bronchoalveolar lavage fluid using enzyme-linked immunosorbent assay (ELISA) kits. The results showed that CBL0137 significantly inhibited the expression of these inflammatory markers, suggesting its potential to mitigate airway remodeling by reducing inflammation (Supporting Information Fig. 1A,B).

### CBL0137 inhibits airway smooth muscle cell proliferation

**Fig. 3:**
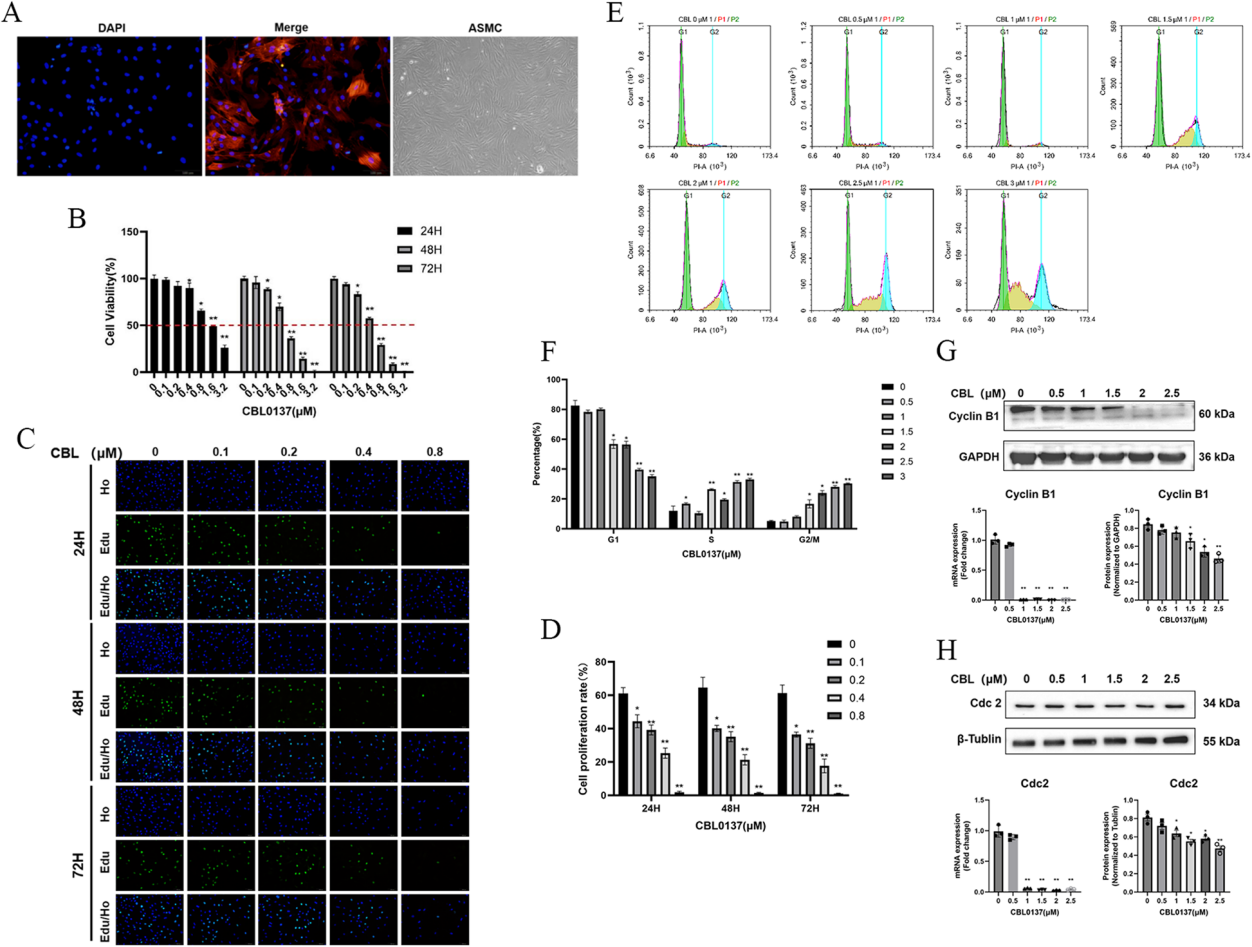
CBL0137 inhibits airway smooth muscle cell proliferation. **A** Immunofluorescence staining of rat ASMCs. **B** Cell viability in rat ASMCs treated with various concentrations of CBL0137 (0, 0.1, 0.2, 0.4, 0.8, 1.6, and 3.2 μM) for 24, 48, and 72 h, measured using the CCK-8 assay. Statistical significance is denoted as ^*^p < 0.05, ^**^p < 0.01 compared with the control group. **C** Proliferation of rat ASMCs after treatment with CBL0137 (0, 0.1, 0.2, 0.4, or 0.8 μM) for 24, 48, or 72 h, measured via the EdU cell proliferation assay. **D** Comparison of the proliferating cell numbers between the treated and nontreated groups. ^*^p < 0.05, ^**^p < 0.01. **E** Flow cytometric analysis of cell cycle distribution in rat ASMCs treated with CBL0137 (0, 0.5, 1, 1.5, 2, 2.5, or 3 μM) for 48 h. **F** Statistical analysis of cell cycle distribution, showing the proportions of cells in G1, S, and G2/M phases. ^*^p < 0.05, ^**^p < 0.01 compared with the nontreated group. **G** Changes in Cyclin B1 transcription and protein expression in rat ASMCs following treatment with CBL0137 (0, 0.5, 1, 1.5, 2, or 2.5 μM) for 48 h, assessed by qPCR and Western blot. ^*^p < 0.05, ^**^p < 0.01 compared with the control group. **H** Changes in Cdc2 transcription and protein expression under the same treatment conditions

The study demonstrated that CBL0137 effectively inhibited airway smooth muscle layer thickening in asthmatic mice, suggesting that it may suppress airway smooth muscle cell (ASMC) proliferation. Immunostaining confirmed that the purity of rat primary ASMCs was nearly 100% (Fig. 3A). The effect of CBL0137 on ASMC viability was assessed using a CCK-8 assay. Results showed a dose- and time-dependent inhibition of cell viability (Fig. 3B), indicating a substantial role of CBL0137 in suppressing ASMC activity. To assess the inhibitory effect of CBL0137 on the proliferation of ASMCs, an EdU assay was performed. The results demonstrated a dose-dependent inhibition of cell proliferation by CBL0137 (Fig. 3C,D), indicating its potent anti-proliferative effect on ASMCs.

Since cell proliferation is tightly regulated by the cell cycle, we next investigated the effects of CBL0137 on cell cycle progression using flow cytometry. ASMCs were treated with varying concentrations of CBL0137 (0, 0.5, 1, 1.5, 2, 2.5, and 3 μM) for 48 h, and cell cycle distribution was analyzed. The results revealed significant changes in the cell cycle following CBL0137 treatment. Specifically, the proportion of cells in the G1 phase decreased, while the proportions in the S and G2/M phases increased in a concentration-dependent manner at concentrations ≥ 1.5 μM. These changes were statistically significant (*p* < 0.05; Fig. 3E and F).

The cell cycle is governed by a series of cyclins and cyclin-dependent kinases (CDKs). As expected, CBL0137 treatment led to a significant reduction in the mRNA levels of Cyclin B1 at concentrations ≥ 1 μM, with a corresponding decrease in Cyclin B1 protein levels observed at ≥ 1.5 μM (*p* < 0.05, Fig. 3G). Additionally, both mRNA and protein levels of Cdc2 decreased in a dose-dependent manner at concentrations ≥ 1 μM compared with the untreated group (*p* < 0.05; Fig. 3H).

### CBL0137 promotes apoptosis in rat ASMCs

**Fig. 4:**
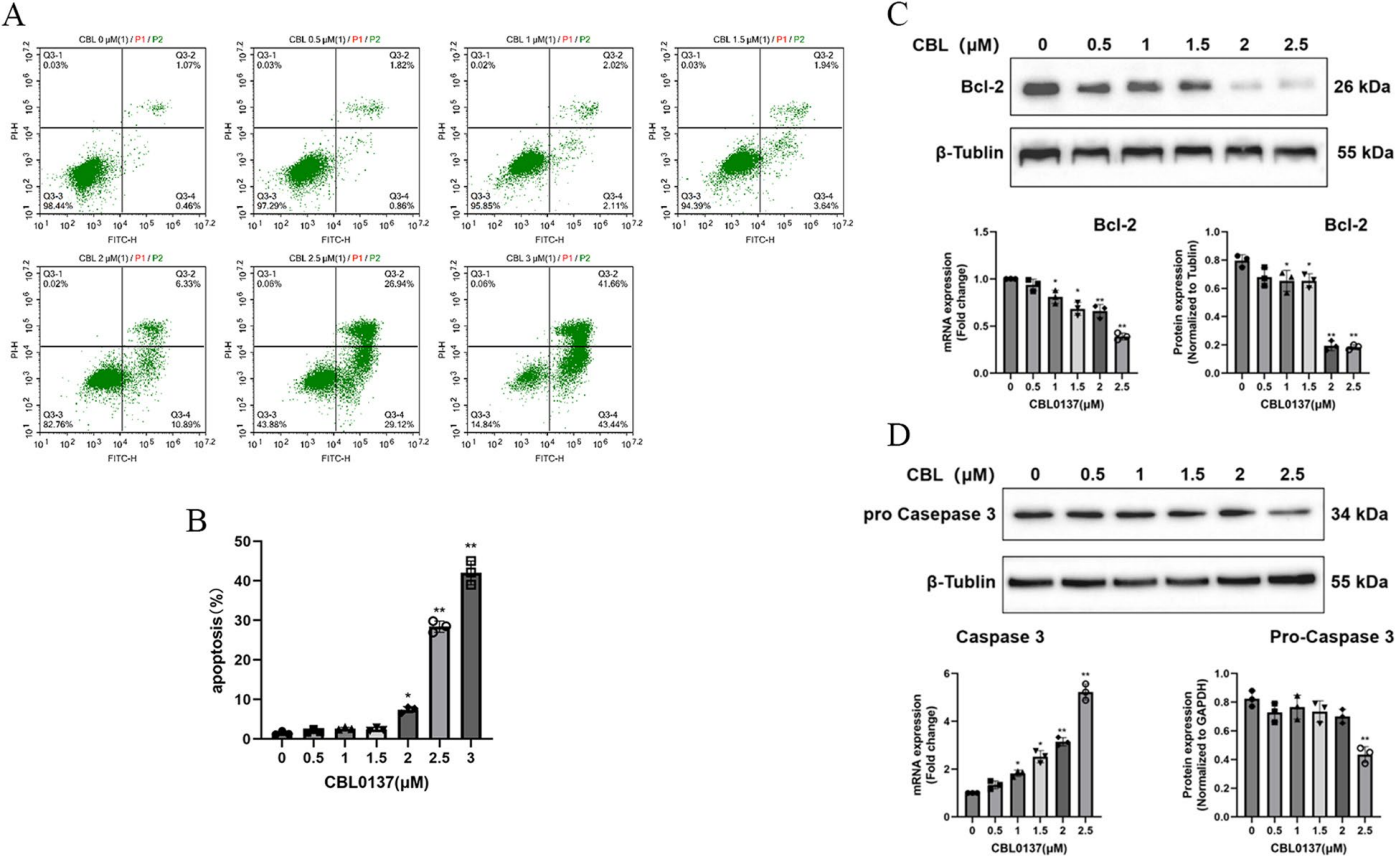
CBL0137 promotes apoptosis in rat ASMCs. **A** After treatment with CBL0137 (0, 0.5, 1, 1.5, 2, 2.5, or 3 μM) for 48 h, the proportion of apoptotic ASMCs was assessed using flow cytometry. **B** Statistical analysis of the proportion of late apoptotic cells. Data were compared with the nontreated group (^*^p < 0.05, ^**^p < 0.01). **C** The expression levels of Bcl-2 mRNA and protein were evaluated in rat ASMCs treated with varying concentrations of CBL0137 (0, 0.5, 1, 1.5, 2, or 2.5 μM) for 48 h (^*^p < 0.05, ^**^p < 0.01). **D** The expression of Caspase-3 mRNA and Pro-Caspase-3 protein was measured after identical treatment conditions (^*^p < 0.05, ^**^p < 0.01)

In addition to regulating cell proliferation, apoptosis plays a crucial role in maintaining the balance of ASMC numbers. To evaluate the effect of CBL0137 on apoptosis in ASMCs, flow cytometry was performed. The results (Fig. 4A) indicated that both early and late apoptosis rates increased with escalating drug concentrations. Notably, the late apoptosis rate was significantly higher at CBL0137 concentrations of 2 μM or greater (*p* < 0.01; Fig. 4B).

To further investigate the effects of CBL0137 on apoptosis-related proteins, Bcl-2 and Caspase-3 mRNA and protein expression were measured by qPCR and Western blot, respectively. At a concentration of 1 μM CBL0137, both Bcl-2 mRNA and protein expression significantly decreased (*p* < 0.05; Fig. 4C). Correspondingly, caspase-3 mRNA expression gradually increased, and Western blot analysis revealed a significant reduction in Pro-Caspase-3 protein expression at a concentration of 2.5 μM (*p* < 0.05; Fig. 4D).

## Discussion

Asthma is a chronic respiratory condition [17], and current treatments primarily focus on controlling inflammation [18, 19]. However, these interventions fail to fully restore lung function or halt disease progression, suggesting that additional factors are involved. One critical pathological feature in individuals with recurrent, chronic asthma is airway remodeling, which plays a role as significant as inflammation in the disease’s pathogenesis [20]. Pathological changes associated with airway remodeling include epithelial hyperplasia of airway astrocytes with dysregulated secretion, collagen fiber deposition, and transformation of the subepithelial basement membrane and periapical alveolar tissue. Additionally, ASM proliferation and contraction, extracellular matrix deposition, and an increase in blood vessel number contribute to airway remodeling [21, 22]. These changes lead to airway hyperresponsiveness, airway obstruction, airflow limitation, and a progressive decline in lung function in asthma patients. As such, targeting inflammation alone is insufficient for optimal clinical management. Studies have shown that glucocorticoids and certain anti-inflammatory drugs reduce inflammation and improve mild to moderate asthma. However, the effects of inhaled or oral glucocorticoids on airway remodeling have not been conclusively demonstrated, with several reports showing conflicting results for inhaled glucocorticoids [23–25]. ASMCs play a central role in airway remodeling by controlling muscle tone, regulating airway lumen opening, and contributing to various pathological changes. These cells are key therapeutic targets, as they are involved in airway hyperresponsiveness, inflammation, and remodeling, which affect asthma progression [26]. CBL0137, a histone FACT inhibitor, has been shown to inhibit the proliferation and promote apoptosis in a range of tumor cells, significantly reducing tumor growth [10, 11]. However, its effects on ASMCs have not been previously reported. If CBL0137 can inhibit ASMC proliferation during airway remodeling, it could be beneficial for treating airway remodeling.

Histological examination using H&E staining revealed significant inflammatory infiltration, endothelial hyperplasia in bronchioles, and smooth muscle layer thickening in the airways, confirming the success of the asthma model and allowing for subsequent experimentation. Lung function tests indicated that as acetylcholine concentration increased, airway resistance slightly increased in the blank control group, with the most pronounced increase observed in the OVA model group, while other conditions remained unchanged. In the asthma model treated with CBL0137 and budesonide, airway resistance was lower than the model group. However, the low-dose (15 mg/kg) CBL0137 group had greater airway resistance than the high-dose (30 mg/kg) CBL0137 group or the budesonide-treated group, with no significant difference between the high-dose CBL0137 group and the budesonide group. Lung function tests are the gold standard for diagnosing chronic airway respiratory diseases [27, 27], commonly used for asthma evaluation. Several parameters in these tests reflect asthma onset and severity, guiding medication usage [28]. Increased airway resistance suggests airway hyperresponsiveness [29], and our results suggest that CBL0137 may alleviate airway hyperresponsiveness in asthmatic mice.

PAS staining showed increased astrocyte numbers [30], which are typically limited in the respiratory epithelium but rapidly proliferate during allergen exposure or inflammation, leading to excess mucus secretion [31]. In comparison to the blank control group, PAS scores in the asthma model treated with CBL0137 and budesonide showed a significant increase in airway epithelial astrocytes. Additionally, both treatments strongly increased collagen fiber deposition, which, when accumulated in lung interstitium, reduces lung elasticity, increases inspiratory resistance, and diminishes ventilation [32, 33]. In this study, collagen fiber deposition was notably higher in the asthma model than the control group, but treatment with CBL0137 and budesonide reduced this deposition. Despite this reduction, collagen fibers still accumulated in the asthma model, with no significant difference between the three treatment groups. H&E staining also showed that the smooth muscle layer of the airways was thicker in the OVA model than in the control group, suggesting ASM hyperplasia. α-SMA immunofluorescence staining revealed significantly higher α-SMA fluorescence intensity in the asthma model than in the control group [34]. However, the fluorescence intensity of α-SMA in the three treatment groups was lower than in the asthma model group, with no significant differences among the treatment groups. Airway remodeling is characterized by epithelial cell damage, increased mucus secretion, and increased ASM mass [35]. These results, together with H&E, PAS, Masson’s trichrome, and α-SMA immunofluorescence staining, suggest that CBL0137 mitigates airway remodeling similarly to budesonide, with no significant difference between high- and low-dose CBL0137 treatments. Notably, lung histopathology indicated that both CBL0137 and budesonide treatments resulted in similar pathological indices to the control group, but lung function tests showed that airway resistance did not fully normalize, implying that additional etiological factors contribute to airway hyperresponsiveness beyond those involved in airway remodeling.

To explore the mechanism by which CBL0137 inhibits airway smooth muscle (ASM) proliferation, ASMCs were isolated from the lung bronchioles of SD rats. Immunofluorescence staining for α-SMA and light microscopy confirmed the high purity of the isolated ASMCs.

To evaluate the effect of CBL0137 on rat ASMCs, we initially conducted CCK-8 assays. These assays revealed that increasing concentrations and prolonged exposure to CBL0137 significantly reduced cell viability, indicating cytotoxicity to rat ASMCs. Given its known inhibitory effect on cell proliferation in cancer models, we further examined the impact of CBL0137 on ASMC proliferation using an EdU assay. The results showed a concentration- and time-dependent decrease in proliferation, with negligible proliferation at 0.8 μM.

Cell proliferation is regulated by the cell cycle, which is controlled by cyclins and CDKs [36]. Disruption of normal cell cycle regulation can impair proliferation, leading to reduced or arrested cell growth [37]. CBL0137 has been reported to block the cell cycle of aggressive B-cell non-Hodgkin lymphoma (B-NHL) cells at the S phase via the c-MYC/p53/p21 pathway, thereby inhibiting proliferation [38, 39]. In this study, flow cytometry revealed that CBL0137 treatment decreased the proportion of G1-phase cells while increasing the percentages of S- and G2/M-phase cells after 48 h of exposure to 1.5 μM of the drug (*p* < 0.05). These findings suggest that CBL0137 arrests the cell cycle at the S and G2/M phases, potentially by modulating cyclins involved in these stages [39]. These findings suggest that CBL0137 arrests the cell cycle at the S and G2/M phases, potentially by modulating cyclins involved in these stages [40]. To investigate this further, we measured the expression of Cyclin-B1 and Cdc2, key regulators of the G2/M phase, using qPCR and Western blotting. CBL0137 significantly decreased both the mRNA and protein levels of Cyclin-B1 and Cdc2 (*p* < 0.05).

In addition to inhibiting ASMC proliferation, CBL0137 promoted apoptosis. Flow cytometry showed a marked increase in late apoptosis when ASMCs were treated with CBL0137 at concentrations of 2 μM or higher. Apoptosis is controlled by two main pathways: intrinsic (regulated by Bcl-2 family proteins) and extrinsic (mediated by tumor necrosis factor (TNF) death receptors). Both pathways converge on the activation of the caspase protease family, resulting in characteristic apoptotic features [41]. Bcl-2, an apoptosis inhibitor, prevents mitochondrial cytochrome C release and inhibits caspase activation [42, 43]. We assessed Bcl-2 and Caspase-3 expression via qPCR and Western blotting. CBL0137 inhibited both mRNA and protein levels of Bcl-2 at concentrations ≥ 1 μM, while it only increased Caspase-3 mRNA expression under the same conditions. At 2.5 μM, Pro-Caspase-3 protein levels were significantly reduced (*p* < 0.05).

## Conclusions

**Fig. 5:**
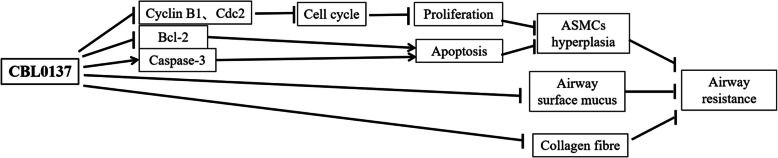
Schematic diagram of the mechanism of CBL0137

In conclusion, CBL0137 alleviates asthmatic airway remodeling and reduces airway resistance by inhibiting airway epithelial astrocyte proliferation, decreasing collagen fiber deposition in lung parenchyma, and preventing thickening of the ASM layer. The mechanisms underlying the inhibition of airway smooth muscle thickening include the suppression of ASMC proliferation and the promotion of ASMC apoptosis. The inhibition of proliferation occurs through downregulation of Cyclin-B1 and Cdc2 expression and cell cycle arrest at the G2/M phase, while apoptosis is promoted via downregulation of Bcl-2 and upregulation of Caspase-3 (Fig. 5).

## Supplementary Information

Supplementary Material 1: Figure S1. CBL0137 reduces asthma-related inflammatory cytokines. (A) Concentrations of IL-4, IL-5, IL-13, IgE, and IgG in mouse lung lavage fluid. Statistical comparisons: ^##^*p* < 0.01 versus the blank control group. ^*^p < 0.05. ^**^p < 0.01 versus the model group. ^&^*p* < 0.05. ^&&^*p* < 0.01 versus the high-dose CBL0137 group compared with the low-dose group. (B) Comparison of serum levels of inflammatory factors related to asthma.

## Data Availability

The datasets used and analyzed during the current study are available from the corresponding author on reasonable request.

## Abbreviations

ASMCs: Airway smooth muscle cells
AHR: Airway hyperreactivity
Bcl-2: B-cell lymphoma-2
CCK-8: Cell Counting Kit-8
Cyclin B1: Cyclin B1
Cdc2: Cell division cycle 2
Caspase 3: Caspase 3
ELISA: Enzyme-linked immunosorbent assay
FBS: Fetal bovine serum
OD: Optical density
PI: Propidium Iodide
PBS: Phosphate-buffered solution
PVDF: Polyvinylidene Fluoride
SPF: Specific pathogen-free
SD: Sprague–Dawley
TBST: Tris Buffered Solution Tween-20
WB: Western Blot
ANOVA: Analysis of variance
ASM: Airway smooth muscle
CDK: Cyclin-dependent kinases
FACT: Facilitate chromatin transcription
GINA: Global Initiative for Asthma
H&E: Hematoxylin and eosin
TNF: Tumor necrosis factor

## References

[1] Nastaravičius A, Ramanauskienė K. Role of a community pharmacy service in care of bronchial asthma patients in Lithuania. Can Respir J. 2018;2018:6060581. 30210645 10.1155/2018/6060581 PMC6120289

[2] Rationale for the use of immunomodulatory therapies in the Global Initiative for Asthma (GINA) step V asthma other than oral glucocorticosteroids - PubMed. https://pubmed.ncbi.nlm.nih.gov/21762333/. Accessed 1 Mar 2024. 10.1111/j.1445-5994.2011.02481.x 21762333

[3] Agusti A, Bel E, Thomas M, Vogelmeier C, Brusselle G, Holgate S, et al. Treatable traits: toward precision medicine of chronic airway diseases. Eur Respir J. 2016;47:410–9. 26828055 10.1183/13993003.01359-2015

[4] Ito T, Hirose K, Nakajima H. Bidirectional roles of IL-22 in the pathogenesis of allergic airway inflammation. Allergol Int. 2019;68:4–8. 30424940 10.1016/j.alit.2018.10.002

[5] Ling K-M, Sutanto EN, Iosifidis T, Kicic-Starcevich E, Looi K, Garratt LW, et al. Reduced transforming growth factor β1 (TGF-β1) in the repair of airway epithelial cells of children with asthma. Respirology. 2016;21:1219–26. 27221564 10.1111/resp.12810

[6] Monocyte-derived fibrocytes induce an inflammatory phenotype in airway smooth muscle cells - PubMed. https://pubmed.ncbi.nlm.nih.gov/25255717/. Accessed 1 Mar 2024. 10.1111/cea.12421 25255717

[7] Effect of icariin on the H2O2-induced proliferation of mouse airway smooth muscle cells through miR-138-5p regulating SIRT1/AMPK/PGC-1α axis - PubMed. https://pubmed.ncbi.nlm.nih.gov/36772811/. Accessed 1 Mar 2024. 10.1177/03946320231151515 PMC9926010 36772811

[8] Pei Q-M, Jiang P, Yang M, Qian X-J, Liu J-B, Zheng H, et al. Upregulation of a disintegrin and metalloproteinase-33 by VEGF in human airway smooth muscle cells: implications for asthma. Cell Cycle. 2016;15:2819–26. 27579513 10.1080/15384101.2016.1220462 PMC5053581

[9] Li R, Wang J, Li R, Zhu F, Xu W, Zha G, et al. ATP/P2X7-NLRP3 axis of dendritic cells participates in the regulation of airway inflammation and hyper-responsiveness in asthma by mediating HMGB1 expression and secretion. Exp Cell Res. 2018;366:1–15. 29545090 10.1016/j.yexcr.2018.03.002

[10] Jin M-Z, Xia B-R, Xu Y, Jin W-L. Curaxin CBL0137 exerts anticancer activity via diverse mechanisms. Front Oncol. 2018;8:598. 30581774 10.3389/fonc.2018.00598 PMC6292929

[11] Albahde MAH, Zhang P, Chen H, Wang W. CBL0137 administration suppresses human hepatocellular carcinoma cells proliferation and induces apoptosis associated with multiple cell death related proteins. Neoplasma. 2020;67:547–56. 32202904 10.4149/neo_2020_190621N535

[12] Yang C, Wang Z-Q, Zhang Z-C, Lou G, Jin W-L. CBL0137 activates ROS/BAX signaling to promote caspase-3/GSDME-dependent pyroptosis in ovarian cancer cells. Biomed Pharmacother. 2023;161:114529. 37002567 10.1016/j.biopha.2023.114529

[13] Burkhart C, Fleyshman D, Kohrn R, Commane M, Garrigan J, Kurbatov V, et al. Curaxin CBL0137 eradicates drug resistant cancer stem cells and potentiates efficacy of gemcitabine in preclinical models of pancreatic cancer. Oncotarget. 2014;5:11038–53. 25402820 10.18632/oncotarget.2701 PMC4294371

[14] Cho KS, Park MK, Kang SA, Park HY, Hong SL, Park HK, et al. Adipose-derived stem cells ameliorate allergic airway inflammation by inducing regulatory T cells in a mouse model of asthma. Mediators Inflamm. 2014;2014:436476. 25246732 10.1155/2014/436476 PMC4160627

[15] Padrid P, Snook S, Finucane T, Shiue P, Cozzi P, Solway J, et al. Persistent airway hyperresponsiveness and histologic alterations after chronic antigen challenge in cats. Am J Respir Crit Care Med. 1995;151:184–93. 7812551 10.1164/ajrccm.151.1.7812551

[16] de Castro LL, Xisto DG, Kitoko JZ, Cruz FF, Olsen PC, Redondo PAG, et al. Human adipose tissue mesenchymal stromal cells and their extracellular vesicles act differentially on lung mechanics and inflammation in experimental allergic asthma. Stem Cell Res Ther. 2017;8:151. 28646903 10.1186/s13287-017-0600-8 PMC5482954

[17] Papi A, Brightling C, Pedersen SE, Reddel HK. Asthma. Lancet. 2018;391:783–800. 29273246 10.1016/S0140-6736(17)33311-1

[18] Kwah JH, Peters AT. Asthma in adults: principles of treatment. Allergy Asthma Proc. 2019;40:396–402. 31690379 10.2500/aap.2019.40.4256

[19] Jones TL, Neville DM, Chauhan AJ. Diagnosis and treatment of severe asthma: a phenotype-based approach. Clin Med (Lond). 2018;18 Suppl 2:s36-40. 29700091 10.7861/clinmedicine.18-2s-s36 PMC6334025

[20] Varricchi G, Ferri S, Pepys J, Poto R, Spadaro G, Nappi E, et al. Biologics and airway remodeling in severe asthma. Allergy. 2022;77:3538–52. 35950646 10.1111/all.15473 PMC10087445

[21] Banno A, Reddy AT, Lakshmi SP, Reddy RC. Bidirectional interaction of airway epithelial remodeling and inflammation in asthma. Clin Sci (Lond). 2020;134:1063–79. 32369100 10.1042/CS20191309

[22] Savin IA, Zen’kova MA, Senkova AV. Bronchial asthma, airway remodeling and lung fibrosis as successive steps of one process. Int J Mol Sci. 2023;24:16042. 38003234 10.3390/ijms242216042 PMC10671561

[23] Wang J, Shang Y-X, Cai X-X, Liu L-Y. Vasoactive intestinal peptide inhibits airway smooth muscle cell proliferation in a mouse model of asthma via the ERK1/2 signaling pathway. Exp Cell Res. 2018;364:168–74. 29408536 10.1016/j.yexcr.2018.01.042

[24] Riccioni G, Di Ilio C, D’Orazio N. Review: pharmacological treatment of airway remodeling: inhaled corticosteroids or antileukotrienes? Ann Clin Lab Sci. 2004;34:138–42. 15228224

[25] Ward C, Reid DW, Orsida BE, Feltis B, Ryan VA, Johns DP, et al. Inter-relationships between airway inflammation, reticular basement membrane thickening and bronchial hyper-reactivity to methacholine in asthma; a systematic bronchoalveolar lavage and airway biopsy analysis. Clin Exp Allergy. 2005;35:1565–71. 16393322 10.1111/j.1365-2222.2005.02365.x

[26] Camoretti-Mercado B, Lockey RF. Airway smooth muscle pathophysiology in asthma. J Allergy Clin Immunol. 2021;147:1983–95. 34092351 10.1016/j.jaci.2021.03.035

[27] Ruppel GL, Enright PL. Pulmonary function testing. Respir Care. 2012;57:165–75. 22222135 10.4187/respcare.01640

[28] Hopp RJ, Wilson M, Pasha MA. A compendium and review of pediatric pulmonary function testing assessment opportunities for asthma. J Asthma. 2022;59:1584–9. 34111364 10.1080/02770903.2021.1941094

[29] Maslan J, Mims JW. What is asthma? Pathophysiology, demographics, and health care costs. Otolaryngol Clin North Am. 2014;47:13–22. 24286675 10.1016/j.otc.2013.09.010

[30] Wolner ZJ, Mustin DE, Stoff BK. Preordering periodic Acid-Schiff staining: a quality improvement study. Am J Dermatopathol. 2023;45:825–7. 37883931 10.1097/DAD.0000000000002570

[31] Birchenough GMH, Johansson MEV, Gustafsson JK, Bergström JH, Hansson GC. New developments in goblet cell mucus secretion and function. Mucosal Immunol. 2015;8:712–9. 25872481 10.1038/mi.2015.32 PMC4631840

[32] Holmes DF, Lu Y, Starborg T, Kadler KE. Collagen fibril assembly and function. Curr Top Dev Biol. 2018;130:107–42. 29853175 10.1016/bs.ctdb.2018.02.004

[33] Geng Y, Li L, Yan J, Liu K, Yang A, Zhang L, et al. PEAR1 regulates expansion of activated fibroblasts and deposition of extracellular matrix in pulmonary fibrosis. Nat Commun. 2022;13:7114. 36402779 10.1038/s41467-022-34870-w PMC9675736

[34] Shinde AV, Humeres C, Frangogiannis NG. The role of α-smooth muscle actin in fibroblast-mediated matrix contraction and remodeling. Biochim Biophys Acta Mol Basis Dis. 2017;1863:298–309. 27825850 10.1016/j.bbadis.2016.11.006 PMC5163362

[35] Liu G, Philp AM, Corte T, Travis MA, Schilter H, Hansbro NG, et al. Therapeutic targets in lung tissue remodelling and fibrosis. Pharmacol Ther. 2021;225:107839. 33774068 10.1016/j.pharmthera.2021.107839

[36] Wang Z. Cell cycle progression and synchronization: an overview. Methods Mol Biol. 2022;2579:3–23. 36045194 10.1007/978-1-0716-2736-5_1

[37] Engeland K. Cell cycle regulation: p53–p21-RB signaling. Cell Death Differ. 2022;29:946–60. 35361964 10.1038/s41418-022-00988-z PMC9090780

[38] Lv Y, Du Y, Li K, Ma X, Wang J, Du T, et al. The FACT-targeted drug CBL0137 enhances the effects of rituximab to inhibit B-cell non-Hodgkin’s lymphoma tumor growth by promoting apoptosis and autophagy. Cell Commun Signal. 2023;21:16. 36691066 10.1186/s12964-022-01031-x PMC9869543

[39] Rieger AM. Flow cytometry and cell cycle analysis: an overview. Methods Mol Biol. 2022;2579:47–57. 36045197 10.1007/978-1-0716-2736-5_4

[40] Clemm von Hohenberg K, Müller S, Schleich S, Meister M, Bohlen J, Hofmann TG, et al. Cyclin B/CDK1 and Cyclin A/CDK2 phosphorylate DENR to promote mitotic protein translation and faithful cell division. Nat Commun. 2022;13:668. 35115540 10.1038/s41467-022-28265-0 PMC8813921

[41] Roberts JZ, Crawford N, Longley DB. The role of ubiquitination in apoptosis and necroptosis. Cell Death Differ. 2022;29:272–84. 34912054 10.1038/s41418-021-00922-9 PMC8817035

[42] Kaloni D, Diepstraten ST, Strasser A, Kelly GL. BCL-2 protein family: attractive targets for cancer therapy. Apoptosis. 2023;28:20–38. 36342579 10.1007/s10495-022-01780-7 PMC9950219

[43] Siddiqui WA, Ahad A, Ahsan H. The mystery of BCL2 family: Bcl-2 proteins and apoptosis: an update. Arch Toxicol. 2015;89:289–317. 25618543 10.1007/s00204-014-1448-7

