# Supplementary figures and images for: Investigation of the mechanistic impact of CBL0137 on airway remodeling in asthma
Source: BMC Pulm Med. 2025 Mar 20;25:129. doi: 10.1186/s12890-025-03596-y (PMC11927260; doi:10.1186/s12890-025-03596-y)

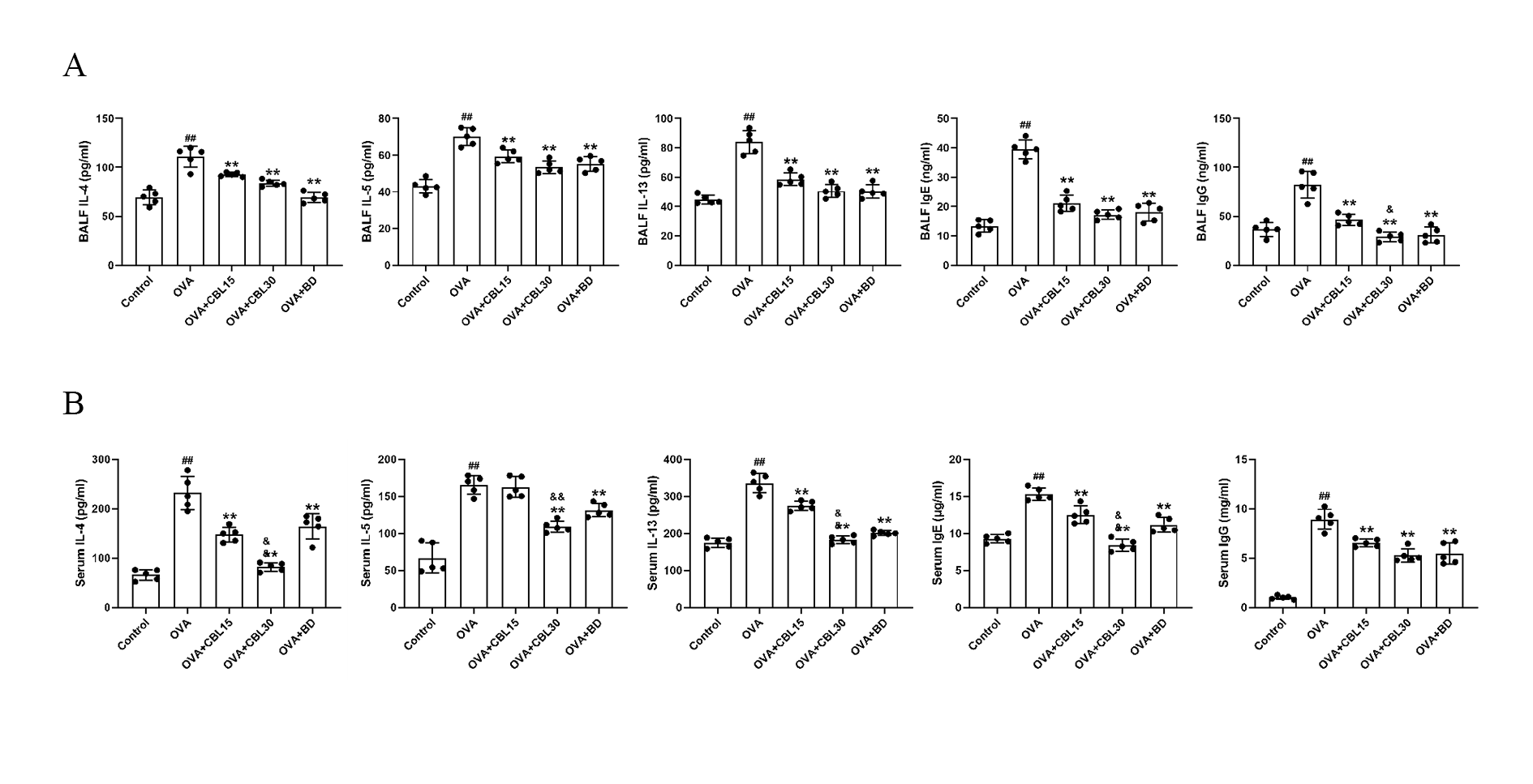

Supplement: Supplementary file 1 — Supplementary Material 1: Figure S1. CBL0137 reduces asthma-related inflammatory cytokines. (A) Concentrations of IL-4, IL-5, IL-13, IgE, and IgG in mouse lung lavage fluid. Statistical comparisons: ##p < 0.01 versus the blank control group. *p < 0.05. **p < 0.01 versus the model group. &p < 0.05. &&p < 0.01 versus the high-dose CBL0137 group compared with the low-dose group. (B) Comparison of serum levels of inflammatory factors related to asthma. [file 12890_2025_3596_MOESM1_ESM.tif]
